# African immigrant parents' understanding of their teenagers' newly diagnosed diabetes status in Perth, Western Australia

**DOI:** 10.1186/1687-9856-2015-S1-P4

**Published:** 2015-04-28

**Authors:** Annette S Hart, Sara Baynes, Sadie Geraghty

**Affiliations:** 1 Princess Margaret Hospital, Perth, WA, Australia; 2 Edith Cowan University, Perth, WA, Australia

## Background

Recently Western Australia has seen a rise in African population due to both economic and refugee migration. Concurrently, a rise in the numbers of teenagers of African origin diagnosed with both type 1 and type 2 diabetes and associated complications has been noticeable. Different ethnic background is a known risk factor for poor metabolic control; this trend is reflected in studies wherein people of African origin have been found to have a high risk of developing diabetes. What is evident from health promotion literature is that parents of teenagers with a chronic health condition, when they are well informed about that condition, play a key part its management. Little is known, though, about what African migrant parents understand about diabetes and its dietary control.

## Aim

To develop insight of a sample of migrant African parents now residing in Western Australia knew about, and were able to provide in relation to the dietary needs of their recently-diagnosed diabetic teenager through an exploration of family food habits.

## Methods

A survey approach.

## Findings

Twelve parents from five different countries of origin participated in this survey. Despite all participants having received education on the topic from a dedicated paediatric diabetes team, it was evident from their families’ food habits that either an understanding of or the capacity to accommodate the dietary requirements of their diabetic teenager was minimal.

## Conclusion

It is possible that African migrant parents of diabetic teenagers’ knowledge about and capacity to support their children’s dietary needs is contributing to unplanned hospital admissions. The results of this small survey indicate a need to revise the information provided to African migrant parents of diabetic teenagers to more closely accommodate cultural preferences. Further work is necessary to determine the most effective approach to health education with this group of health care consumers.

